# Study on the logic and effectiveness of crisis learning in the promotion policy adjustment: an observation based on the adjustment of COVID-19 prevention policy in China

**DOI:** 10.3389/fpubh.2023.1324420

**Published:** 2024-01-05

**Authors:** Changwei Wei, Jiaxi Xu, Zuying Xu

**Affiliations:** ^1^School of Public Policy and Management, China University of Mining and Technology, Xuzhou, China; ^2^School of Political Science and Public Administration, Wuhan University, Wuhan, China; ^3^School of Economics and Management, Huaibei Normal University, Huaibei, China

**Keywords:** crisis learning, policy adjustment, COVID-19 prevention policy, policy system, emergencies

## Abstract

**Background:**

As the impact of COVID-19 on normal production and living conditions diminishes, this serious emergency is come to an end. China’s policy framework has facilitated positive adjustment over the past 3 years by timely modifying its emergency response to changes in viruses and epidemics. This paper aims to explore the logic of China’s policy framework that promoted policy adjustment through crisis learning during COVID-19.

**Methods:**

By gathering and classifying China’s epidemic prevention policies throughout the past 3 years, integrating policy texts, and analyzing key events, this article examines the process of supporting policy adjustment through crisis learning in the policy system during COVID-19.

**Results:**

The Chinese government’s COVID-19 policy adjustment process can be divided into four stages, namely ‘The period of stress response’, ‘The period of COVID-19 prevention and control’, ‘The period of regular prevention and control’, and ‘The period of overall adjustment’. The policy adjustments in each stage demonstrate the logic and effectiveness of crisis learning in the promotion policy adjustment. The study has determined that the motivational logic comprises three crucial elements: security requirements, accountability pressure, and reputation management. The institutional logic encompasses both the organizational and resourceful environments, and the institutional and cultural environment. Additionally, the behavioral logic of policy adaptation aligns with the strategy of crisis learning. Meanwhile, the logical framework of ‘crisis learning-policy adjustment’ can be verified using the Chinese government’s policy adjustment in COVID-19 as an example.

**Conclusion:**

Establishing an effective post-crisis learning system is crucial to improving the effectiveness of crisis response. There is a logical link between crisis learning and policy adjustment. The implementation of policy adjustment needs to be based on the results of crisis learning. Government departments are essential for crisis learning and policy adjustment.

## 1 Introduction

Since the beginning of 2020, the Chinese government has implemented substantial policies to control COVID-19. Looking at the policy documents issued by the China’s National Health Commission (NHC), we can find that COVID-19 has raised an intricate policy question. The occurrence of clustered infection at different times causing the epidemic to increase the complexity of the emergency response (1). Looking back at China’s anti-epidemic policy, it is clear that as soon as COVID-19 broke out, the Chinese government promptly initiated an emergency response, implemented measures in response to the situation, and persistently issued new policies while adjusting policy directions after many outbreaks. Finally, The NHC is managing COVD-19 with measures against Class B infectious diseases from January 8, 2023, after nearly 3 years of epidemic control (2). It can be seen that the Chinese government has relied on learning from the crisis, acquiring information and knowledge from the epidemic, and implementing policy adjustments as the primary policy measures to manage the COVID-19 outbreak.

Crisis learning is learning from crises (disasters, accidents, emergencies, etc.). Through crisis learning, decision-makers can ‘reflect on actions taken, retain those procedures that proved effective, and discard those that did not’ (3). Various strands of research in the policy sciences have recognized that learning plays a critical role in our ability to understand, influence, and address complex policy issues. Learning can bring new issues to light, challenge previously held beliefs, and help identify innovative policy responses (4). Existing studies on crisis learning cover a wide range of fields. Deverell (5) proposed ‘Crisis Induced Learning’, Antonacopoulou (6) proposed ‘Learning in Crisis’. Moynihan (7) proposed conceptual and defining studies such as ‘Intercrisis Learning’ and ‘Intracrisis Learning’. Elliott (8) constructed the organizational crisis learning process based on the knowledge management perspective and believed that organizational crisis learning is a cyclical process. Drupsteen et al. (9) focused on a specific processing link in crisis learning from a systematic perspective and starting from the reflection of lessons learned from crises. Stern (10) identified the impact of crisis learning on government organizations and the influencing factors of crisis learning from both positive and negative perspectives. Kim et al. (11) explored how to promote the process of crisis learning and how to achieve dual-loop crisis learning. These studies describe the initiation, progress, and outcome of crisis learning, which serves as the foundation for our research.

The proposal of policy adjustment is based on the process of public policy making and implementation. Decision-makers have to adapt to changes in the environment (12), the past policies (13), and the uncertainties about the future (14). The policy-making process is often characterized by limited rational decision-making due to lack of information, insufficient decision-making capacity and decision-making resources (15). Decision-makers need to change the way they define their goals in a complex, dynamic environment (16), and the existence of a crisis situation happens to dramatically changes the social environment. Riggs et al. (17) pointed out that crisis is necessary to promote policy change. A crisis impacts and damages the normal economic and social environment and to some extent creates opportunities for organizational, institutional and policy change. Therefore, it is sometimes seen as a ‘trigger for reform, a promoter of change, or an opportunity for learning’ (18–20).

The above studies focused on crisis learning and policy adjustment as separate areas, without thoroughly integrating them. Although some scholars have proposed that crises can promote policy adjustment, there has been limited research on the combination of crisis learning and policy adjustment. Based on reality, it is clear that policy adjustment is carried out gradually in the process of policy operation, because real conditions have changed. It is essential for decision-makers to make objective policy adjustments based on their comprehension of the decision-making environment and the knowledge and experience they have acquired. We also found that some scholars have conducted retrospective studies (21) and comparative studies (22) on the Chinese government’s COVID-19 policy. However, the combination of the Chinese government’s crisis learning behavior and policy adjustment process in response to the COVID-19 remains unexplored.

This study combines crisis learning theory with policy adjustment behavior for the first time by examining the Chinese government’s policy texts in COVID-19. Through the identification of key events, we separated the Chinese government’s epidemic prevention approach into four stages. Then, the high-frequency words that appeared in the policy texts of each stage were used to identify the focus of the policies. Combining with the judgment and observation of this typical case, it is possible to link the crisis learning behaviors to the logic of policy adjustment. This study can also contribute to the explore the effectiveness of crisis learning in the promotion policy adjustment.

## 2 Methods

### 2.1 Research design and data collection

As a national public health policy-making agency, the NHC provides the highest guidance for prevention and control of COVID-19 in China, and its policies reflect the direction of decision-making. We utilized software to capture 237 policy texts by searching the NHC website for publicly available policy documents from January 20, 2020 to January 8, 2023 (the time period was determined by analyzing China’s policy direction). Since the disease outbreak, the NHC has issued announcements, notices, recommendations, and normative documents, as well as national policy documents on epidemic prevention and control. They include the strategies for epidemic prevention and control, the diagnosis and treatment protocol, and other related matters. Covering a broad range of social activities and aspects, these policies reflect various policy decisions made by the Chinese government at different times and consider specific requirements for particular areas. We divide the policy adjustment over the past 3 years into four stages based on the gradual characteristics of policy adjustment: stress response (before January 20, 2020), COVID-19 prevention and control (from January 20, 2020 to April 29, 2020), regular prevention and control (from April 29, 2020 to November 10, 2022), and overall adjustment (from November 10, 2022 to present).

### 2.2 Analysis and processing

We selected 237 policy texts on epidemic prevention from January 20, 2020 to January 8, 2023 and sorted them by comparison and deduplication. After dividing the policy adjustment process into stages, we decided to analyze the characteristics of the policy texts. We extracted the 40 words with the highest frequency of occurrence in the policy texts, removed meaningless nouns, and then we identified the 16 keywords with the highest word frequency (Table 1). The following observation of the adjustment of the COVID-19 prevention policy can be more effectively supported by structuring the list of high-frequency keywords.

**Table 1:** High-frequency words in COVID-19 prevention policy texts at different stages.

Rank	The period of COVID-19 prevention and control		The period of regular prevention and control		The period of overall adjustment	
	Keywords	F	Keywords	F	Keywords	F
1	COVID-19^***^	1,717	Nucleic acid testing^**^	668	COVID-19^***^	397
2	Prevention and control^**^	1,249	Prevention and control^**^	631	Infection^*^	313
3	Medical^***^	736	COVID-19^***^	581	Medical^***^	306
4	Services^***^	662	Disinfection^**^	462	Joint epidemic prevention and control^**^	297
5	Patients^**^	631	Medical^***^	407	Hospital^**^	269
6	Community^*^	610	Services^***^	357	Treatment^*^	247
7	Isolation^*^	453	Health^***^	313	Services^***^	225
8	Protection^**^	446	Safety^*^	266	Virus^**^	172
9	Disinfection^**^	428	Patients^**^	266	Nucleic acid testing^**^	147
10	Psychology^*^	426	Hygiene^**^	256	Health^***^	139
11	Medical personnel^*^	383	Laboratory^*^	225	Antigen testing^*^	128
12	Health^***^	381	Protection^**^	208	Severe case^*^	123
13	Hygiene^**^	358	Unit^*^	170	Home quarantine^*^	108
14	Cases of disease^*^	283	Risks^*^	165	Grass roots^**^	106
15	Hospital^**^	259	Joint epidemic prevention and control^**^	161	Vaccination^*^	87
16	Grass roots^**^	215	Virus^**^	148	Seniors^*^	86

*** Words that appear in the list of high frequency words in all three stages.

** Words that appear in the list of high-frequency words in two of the stages.

* Words that first appear in the list of high frequency words at this stage.

**Figure 1:**
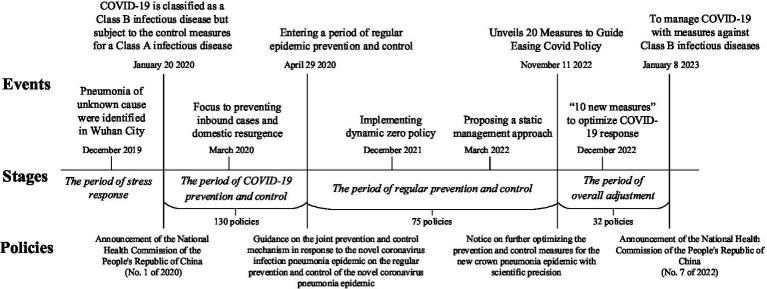
Classification of adjustment stages and documents of COVID-19 prevention policies.

In addition, we use the publication of policies and the selection of key events to construct a timeline of policy adjustments in response to the epidemic (Figure 1), which better confirms the logic and effectiveness of the crisis learning in the promotion policy adjustment from the observation of reality.

## 3 Results

### 3.1 Model construction

**Figure 2:**
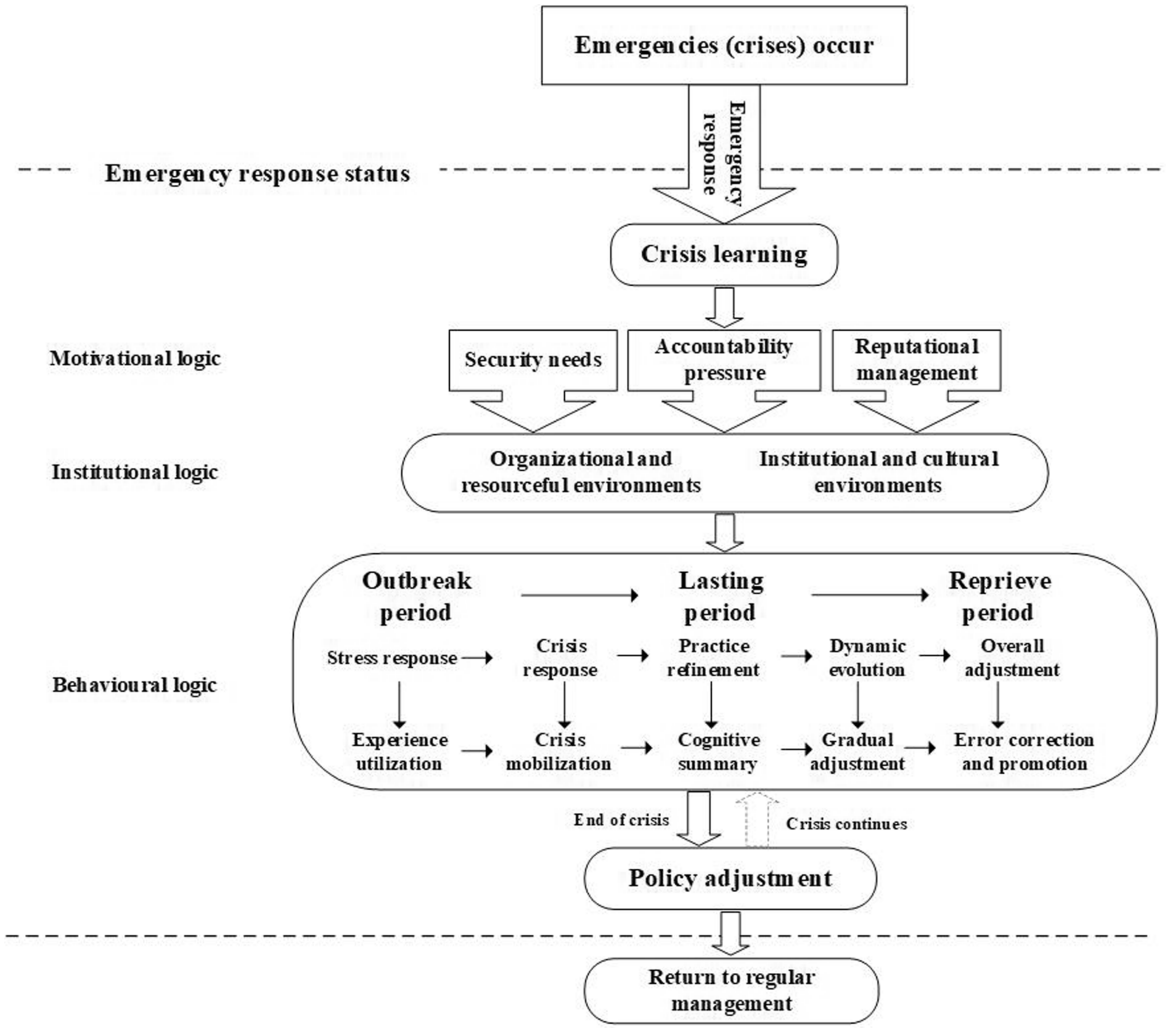
Logic diagram of crisis learning promoting policy adjustment.

As shown in Table 1, the NHC issued 237 policies with a strong emphasis on COVID-19 prevention and control in a short period of time. These documents provided policy recommendations on multiple areas. Changes in high-frequency words in policy texts are a visual embodiment of policy adjustments that reflect the intrinsic behavioral logic of the government. The government is a rational organization, and the emergence of its behavior is linked to the specific social environment and institutional environment. It is reasonable and feasible to observe the government’s motivational and institutional logic through the policy adjustments. Figure 1 demonstrates a correlation between the Chinese government’s response to COVID-19 and policy. It can be seen that there is a logical link between crisis learning behaviors and policy adjustment. Through analysis of the aforementioned data, the internal logic of policy adjustment during crisis learning has been clarified. The process of crisis learning for promoting policy adjustment follows the ‘motivation-institution-behavior’ logic: the motivation for crisis learning addresses the question of why policy adjustment is taking place; the institutional and cultural environment of the crisis learning serves as the foundation for policy adjustment; and the crisis learning strategy directs the path of the policy adjustment. The interconnection between the three logics creates a comprehensive and logical chain that illustrates the process of crisis learning that promotes policy adjustment (Figure 2).

#### 3.1.1 The triple logic of crisis learning in the promotion policy adjustment

##### 3.1.1.1 Motivational logic

Crisis learning is the logical starting point for why the policy system ought to take measures for policy adjustment.

First, crises disrupt social and organizational practices (23). The public’s desire for ‘safety’ is to recover as soon as possible from the crisis and to seek a safe, peaceful, and happy living environment. The public expects the government to be prepared and ready to deal with crises because they understand how these factors contribute to effective crisis management (24). At the same time, the development of Internet media and the public’s voice on the Internet have also forced the government to pay attention to public opinion.

Second, a hierarchical bureaucracy can still be present in the contemporary emergency management, even in the exceedingly dangerous environment of emergencies (25). Crisis generate enormous political pressure (26), and top-down accountability pressures can also encourage crisis learning in the policy system. Policy adjustment can also describe the proactive measures taken by the government to respond to crises.

Finally, the crisis has exposed the problems of the crisis emergency management system. The failure of policy can also stimulate a reconsideration of the existing dominant causal reasoning about policy, potentially leading to crisis learning (27). Unreasonable, untimely, and unscientific emergency behaviors and responses during crisis are highly probable to damage the government reputation, especially in the social environment of the rapid development of the Internet, where minor failures of the government emergency behavior may experience magnified pressure from Internet activism and more intense social comparison (28).

##### 3.1.1.2 Institutional logic

“The belief systems and associated practices that dominate the organizational field” are referred to as institutional logic. Such logic creates institutions and organizations, as well as structured interactions between organizational actors, which are held together by a shared set of meanings, as conceived by institutional logic (29). According to organizational sociology, the organizational and resourceful environment, together with the institutional and cultural environment, can serve as the institutional logic that explains the occurrence of policy system behaviors (30).

In terms of observing the organizational and resourceful environment, crisis learning should be developed with the emergency management department as the core. The collaborative engagement of multiple government departments and social groups to establish an effective combination of the comprehensive management department’s command capacity and the specialized departments’ professional research capacity (31). At the same time, the outbreak of the crisis prompted the government to take strict measures to control the situation. However, many industries were impacted, with reduced production, mobility constraints, and a compressed timeline that forced the space and time for crisis learning, and the quality of policy adjustment was also influenced by the quality of crisis learning.

The ‘One Planning plus Three Systems’ provides a significant legal assurance for the response to disasters and the growth of crisis learning in China, according to observations of the institutional and cultural context. China’s strong degree of politicization may accelerate the concentration of learning resources, but it may also lead to problems such as over-intervention and overreach in the policy adjustment process. Policy adjustment is more politicized when it is influenced by political factors. Traditional cultural beliefs such as ‘Man will conquer nature’, ‘Human effort is the decisive factor’, and ‘Put people’s safety and health as the top priority’ can also influence policy.

##### 3.1.1.3 Behavioral logic

The key principle for policy adjustment in decision-making systems is the crisis learning method, which is also be modified according to the evolution of the crisis.

The policy system still requires a better understanding of the problem in the early stages. Rapid and substantial action must be taken to control the outbreak with limited information and many uncertainties (32). Therefore, the most effective crisis learning strategy is to repeat previous routines (33), combined with empirical policy adjustment paths. When a new crisis exceeds the scope of previous recognition and handling procedures, the policy system must modify the learning mechanism based on the new characteristics of the crisis. After a period of response and disposition, the policy system eventually gathers certain aspects of the problem and should make cognitive summary policy changes. At the same time, the enormous duration and period of the crisis must be taken into account, and the dynamic evolution of crisis learning leads the policy system to deal with the problems brought by the ever-changing crisis through a gradually modified path. With the development of crisis learning, decision-makers have more opportunities and space to thoroughly evaluate, correct, and improve deficiencies in the crisis response process.

The sustainable and effective learning outcomes from gradually deepening crisis learning and the policy adjustment approaches can be classified into the following types (Table 2).

**Table 2:** The strategies of crisis learning and the paths of policy adjustment in different stages.

Stages	Outbreak period		Lasting period		Reprieve period
Strategies for crisis learning	Stress response	Crisis response	Practice refinement	Dynamic evolution	Overall reconfiguration
The path to policy adjustment	Experience utilization	Crisis mobilization	Cognitive summary	Gradual adjustment	Error correction and promotion

#### 3.1.2 The effectiveness of crisis learning in the promotion policy adjustment

Policy adjustment is the result presented by the emergency management system through crisis learning. Through the logical analysis of ‘motivation-institution-behavior’, the logic of crisis learning in the promotion policy adjustment has been clarified. Policy adjustment is the result of the policy system operating according to the logic described above.

##### 3.1.2.1 Adjustment direction based on motivational logic

The direction of policy adjustment is determined by the logic of motivation. Thus, the triple motivation of crisis learning should first be examined to determine the point of adjustment.

The people-centered development philosophy addresses the core issues in China’s governance. In the face of the public’s requirement for safety, policy adjustment consistently adheres to the people-centered value orientation. Protecting the public’s life, health and safety is the cornerstone of policy adjustment.

In the face of post-crisis accountability pressures, policy adjustment should maximize the effectiveness of the accountability mechanism in the hope of improving the performance of public officials during emergencies. COVID-19 exposed an unexpected problem of the accountability system. Instead of incentivizing public officials to take responsibility, it may have discouraged them from making timely, but potentially risky, decisions. There is an urgent need for a well-designed error-tolerance mechanism that can differentiate between tolerable and punishable errors and thus encourage proactive action (34).

In response to the demands of government reputation, policy adjustments aim to strengthen the government reputation management and cultivate the government’s positive image; the government’s effectiveness in crisis management will be enhanced by successful reputation management and communication with the public (35).

##### 3.1.2.2 Adjustment environment based on institutional logic

In the stage of crisis response, the policy system must restructure the original policy mechanism in accordance with the logic of motivation. However, the policy adjustment is influenced by the organizational and resourceful environment, as well as the institutional and cultural environments, and the policy adjustment environment can be constructed through the institutional logic of crisis learning.

In terms of the organizational and resourceful environment, the response to major crisis typically consists of central ministries and commissions collaborating with the emergency management department to form a crisis response leading group, linking various functional departments to provide services such as medical treatment, material protection, transportation, and so on (36). As the crisis evolves, the coordinated emergency response mechanism of emergency based on local government should meet the needs of responding to emergencies in dynamic risk management by strengthening the resilience of the emergency management system.

In terms of the institutional and cultural environment, the enormous impact of the crisis has exposed the weakest part of the institutional system (37). The government’s response to the crisis should be based on improving the emergency response mechanism within the framework of legal governance and developing a crisis response policy system from the very beginning of the crisis in accordance with current laws. At the same time, the policy program must be adjusted to reflect the new circumstances.

##### 3.1.2.3 Adjustment paths based on behavioral logic

Policy adjustment is always problem-focused, responsive to the situation and revised on the basis of the crisis learning strategy.

It is crucial for the policy system to manage crises by identifying the main stages of crisis learning. This involves identifying the actual needs and lessons of crisis learning, revising outdated policies, improving the scientific aspects of policies and increasing the effectiveness of their implementation. This ensures the stability of the policy and the direction and accuracy of its content.

If the policy adjustment fails to prevent the crisis from spreading, policy adjustment at this stage should be based on a crisis learning approach. This approach emphasizes two main elements: ‘dynamism’ and ‘improvement’ while continuously reforming and improving the response method in the continuous crisis response. The adjustment of policy resulting from crisis learning is vital for enhancing organizational resilience. There is a transformation of crisis learning into policy adjustment practice, which then leads to the modification of policy attempts and tools. When governments make such policy adjustments, crisis situations can be effectively prevented from causing further damage and impacting on social institutions.

The ‘crisis learning-policy adjustment’ framework is an analytical framework with some abstract meaning, derived from the mining of policy texts and key events. To validate the interpretability of the framework, we will conduct a practical test on the policy adjustments implemented by the Chinese government during COVID-19.

### 3.2 Practical test

#### 3.2.1 The period of COVID-19 prevention and control: policy adjustment led by experiential learning

##### 3.2.1.1 Stress response: experience utilization

The Wuhan city government arranged for experts to look into these cases through an analysis of the patients’ condition and clinical outcome, the findings of epidemiological investigations, and preliminary laboratory testing results in December 2019 and issued a notice (38). COVID-19 originally caught people’s attention (39). On January 1, 2021, the NHC set up a leading group on the disease response. Wuhan City Health Commission (WCHC) changed ‘viral pneumonia of unknown cause’ to ‘pneumonia caused by the novel coronavirus’ in an information circular on January 12. In the middle of the night of January 19, after careful examination and deliberation, the team determined that the new coronavirus was spreading between humans (40).

COVID-19 is a highly contagious infectious respiratory disease caused by a novel virus. It is difficult to prevent and control, has multiple modes of transmission, and is poorly understood (41); Society had already experienced feelings of uncertainty, concern and even fear at the beginning of the epidemic. As the epidemic spread and the number of cases increased, so did the questions and suspicions (42). Panic and rumors spread quickly on social media and the Internet, which has created chaotic social situations (43).

Crisis learning at this stage is typical of stress-responsive learning. In the case of such novel diseases, knowledge about the nature of the problem and the best ways to address it was particularly inadequate at the outset, as much about the disease and potential solutions to its virulence and spread was poorly understood (44). In order to control the development of the crisis in a short period of time, the fastest and most effective response strategy is to use existing experience for crisis learning and rapid response during the crisis.

At this stage, policy adjustment mainly relies on experience. Based on the emergency management framework and the lessons learned from the SARS epidemic, the policy system responded effectively, taking appropriate measures to separate patients and preventing the spread of the virus. Concurrently, the propaganda department actively publicizes the latest epidemic progress to the public, easing social tensions and seeking public cooperation in implementing epidemic prevention measures.

##### 3.2.1.2 Crisis response: crisis mobilization

On January 20, the NHC made a statement on bringing the pneumonia under quarantinable infectious disease management in accordance with the Frontier Health and Quarantine Law of the People’s Republic of China. NHC also released Protocol on Prevention and Control of Novel Coronavirus Pneumonia. Wuhan City Novel Coronavirus Prevention and Control Command Center issued the No. 1 public notice declaring temporary closure of the city’s outbound routes at its airports and railway stations at 10 a.m. (45).

**Figure 3:**
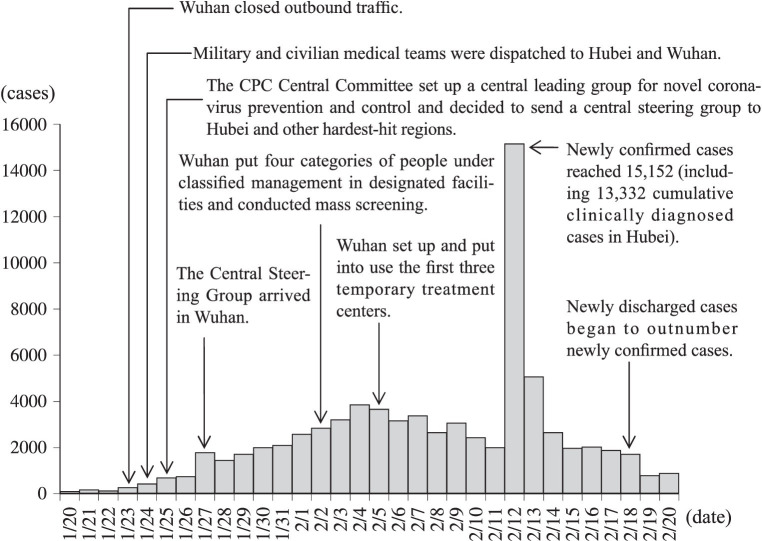
Daily figure for newly confirmed cases on the Chinese mainland (January 20–February 20).

At this point, new confirmed cases in China are fast increasing (Figure 3), and the epidemic is affecting a wide range of businesses. The scarcity of medical resources has put an additional strain on epidemic prevention, and the growing number of cases and deaths has cast a shadow over the entire society. At the same time, a group of government officials, such as Zhu Baohua of the Hubei Provincial Market Supervision and Administration Bureau and Tang Zhihong of the Huanggang Health Commission, were investigated and held accountable for ineffective epidemic prevention measures, providing a strong deterrent to government officials.

At this stage, the crisis learning strategy is quick crisis response. Eradicating and responding to the impacts of the epidemic became the focus of the crisis response. In order to cope with the complexity, fragmentation, uncertainty of the crisis, the decision-making system immediately launched an emergency response, and society entered a state of emergency.

The policy system fully mobilizes social forces to participate in epidemic prevention and control through three crisis mobilization measures: Party and government mobilization, market mobilization, and social mobilization (46). To alleviate the tense situation of epidemic prevention, the Chinese government has set up temporary hospitals, restricted personnel mobility, isolated close contact patients, and suspended public transportation (47). In response to the shortage of epidemic prevention materials and the decline in medical capacity, the policy system is making full use of its institutional advantages to pool medical resources from across the country. At the same time, a number of policies was released to encourage the restart of work (48). In response to the pressure of accountability in difficult times, party and government agencies have also stimulated officials’ enthusiasm through organizational incentives (49).

The focus of policy adjustment is on the directions of ‘COVID-19’, ‘prevention and control’, ‘medical’, ‘service’, and ‘patients’, with the primary objective of saving lives, curing patients, improving cure rates, and reducing mortality rates. Meanwhile, based on experience and learning, the policy system has adopted measures such as ‘isolation’, ‘protection’ and ‘disinfection’ to effectively prevent the spread of the pandemic. Keywords like ‘psychology’ and ‘medical personnel’ emphasize the need to pay attention to the mental health of medical personnel. The policy system broadens the policy focus to include mental health care (50).

#### 3.2.2 The period of regular prevention and control: policy adjustment under the guidance of cognitive learning

##### 3.2.2.1 Refining the practice: summarizing experience

Xi Jinping chaired a meeting of the Standing Committee of the Political Bureau of the Communist Party of China (CPC) Central Committee. He concluded that thanks to arduous efforts, China had won a vital battle in defending Wuhan and Hubei against the novel coronavirus, and achieved a major strategic success in the nationwide control efforts (51).

Crisis learning at this stage is practice-refining learning. Containment strategies that shielded the susceptible population from the transmission process were quite effective—compared with the potential number of cases in an unmitigated outbreak, only a small fraction of the Chinese population at risk was infected (52). Public life is gradually recovering and, assuming the outbreak situation is stable, an orderly return to normal economic and social activity is expected.

Policy adjustment focuses on learning from the crisis and follows a cognitive summary-driven adjustment path. Local governments have taken a series of measures to stabilize employment, safeguard market transactions, and strengthen financial support to promote the orderly resumption of work by enterprises and the gradual recovery of society. Prevention and control of the epidemic continue to focus on promoting the scientific use of masks (53), increasing ventilation and disinfection, and raising awareness of epidemic prevention. The global vaccine R&D effort in response to the COVID-19 pandemic is unprecedented in scale and speed. Additional vaccine development efforts have been undertaken in China (54).

##### 3.2.2.2 Dynamic evolution: gradual adjustment

The Chinese government first adopted the concept of a ‘dynamic zero COVID-19 strategy’ in December 2021, which refers to a thorough maximum prevention and control effect while minimizing social costs by dealing with sporadic cases and clustered epidemics with relative speed and efficiency (55). Shanghai used ‘static management’ for the first time in March 2022 to eliminate potentially contaminated people as early as possible through strict control methods (56).

At this stage, crisis learning is dynamic and evolutionary, adapting to the changing circumstances of the pandemic. It explores more balanced and rational prevention and control measures through continuous dynamic learning. As the worldwide epidemic continues to spread, the domestic local aggregated epidemic has begun to exhibit the characteristics of ‘many points, wide surface, and frequent’. The prevention and control situation is grim and complex (57). The spread of the disease in many regions has increased people’s fear of crisis. However, in many parts of the country, prolonged and frequent ‘nucleic acid testing’ and the ‘dynamic zero COVID-19 strategy’ have overwhelmed the health system, putting enormous strain on health resources and increasing public concern about the government’s efforts to prevent and control the epidemic.

Based on the dynamic crisis learning strategy, policy adjustments were implemented gradually, with the ‘omicron’ strain emerging as the primary culprit after March 2022 (58). Government agencies have improved vaccination strategies and promoted immunization for children and the older adult. Through a series of regulations, it promoted the concept of ‘early detection, early isolation, early diagnosis and early treatment’ and standardized epidemic prevention and control strategies.

‘Prevention and control’ and ‘COVID-19’ remain at the forefront of the policy-making process. The use of ‘nucleic acid testing’ to identify potential sources of risk has become a powerful tool for governments to deal with outbreaks; this is accompanied by the emergence of high-frequency words ‘laboratory’ to strengthen the standardization of laboratory processes and standardized operations is also a new feature of this stage. The use of words such as ‘risks’, ‘joint epidemic prevention and control’ and ‘unit’ illustrates a change in policy focus, emphasizing multi-sectoral coordination and linkage, as well as rigorous prevention and control at the grassroots level.

#### 3.2.3 The period of overall adjustment: reconstructing policy adjustment under the leadership of learning

On November 10, the Standing Committee of the Central Political Bureau held a meeting to discuss the current pandemic situation in China. Twenty measures were officially announced to further optimize the COVID-19 response (59). On November 11, 2022, the Chinese government published the Scientific and Targeted Prevention and Control Measures to Optimize COVID-19 Response (60), and the optimization of prevention and control measures has become a clear policy direction.

This stage of crisis learning is distinguished by overall adjustment, a comprehensive reconstruction type of learning from the previous phase. Vaccination rates have risen steadily, and preparedness for epidemics have improved significantly. During the 3 years of fighting the epidemic, emergency management has gained a wealth of experience. The medical system’s ability to treat patients, detect pathogens, and conduct epidemiological studies has improved (61). At the same time, the public reacted strongly to the government’s over-preparedness for the epidemic as it continued.

Based on the foundation of crisis learning, the adjustment of the policy system at this stage was mainly aimed at correcting and improving the mistakes. Optimization measures such as the ‘20-point measures’ and the ‘10-point measures’ were introduced to optimize epidemic strategies such as cross-border mobility. The NHC has uncovered the more frequent and concentrated epidemic-related problems that the public is aware of. The relevant ministries have also taken considerable responsibility, in accordance with the law, for unjustified epidemic prevention practices, such as charging for quarantine areas, arbitrary silence, and sealing off cities rather than allowing local governments to control them.

The learning adjustments in this stage were built on the foundations of the previous stage, and policy reconstruction and innovation were undertaken in response to the epidemic dynamics of the current situation. The term ‘infection’ replaced ‘patients’ as the focus of policy attention during this phase, and the introduction of the terms ‘antigen testing’ and ‘home quarantine’ shows the adjustment of measures for mild illness to better epidemic prevention once the virus’ pathogenicity has decreased (62). The terms ‘medical’ and ‘hospital’ have continued to receive attention and focus, with policy attention continuing to focus on the lack of overall health resources and the imbalance in regional health resources. The words ‘virus’, ‘services’, ‘health’, ‘treatment’ and ‘vaccination’ emphasize the importance of the new policy. The use of the terms ‘older adult’ and ‘severe case’ demonstrates the policy’s concern for vulnerable people. These terms indicate the context of the new pandemic, which is not yet over, and optimize public health protection through increased vaccination.

In brief, China’s policy for managing and preventing epidemics has gone through a gradual and ongoing process of change, adapting to present circumstances. The analysis has revealed that there is value in recognizing the presence of ‘no change’ in the midst of change, and that the inception of policy adjustments regarding epidemic control and prevention consistently prioritizes public health. At the same time, the Chinese government has taken the initiative to adjust their policies to COVID-19 while ensuring policy stability. In COVID-19, the Chinese government prioritized safeguarding public health and minimizing the impact of the outbreak on economic and social progress.

## 4 Conclusion and discussion

### 4.1 Key results

This paper outlines the Chinese government’s anti-epidemic policy from the past 3 years, dividing it into four stages. We analyze the crisis learning process and policy adjustment practices in each stage. We analyze the crisis learning process and policy adjustment practices within each stage to establish a framework for ‘crisis learning-policy adjustment’, which focuses on developing a logic of crisis learning in the promotion policy adjustment. Our analysis demonstrates that: (1) The motivations driving policy adjustments in crisis learning are mainly security needs, accountability pressure, and reputation management. (2) The organizational and resourceful environments, institutional and cultural environments based on crisis learning are the scenarios in which policy adjustment is carried out; (3) strategies such as stress response, crisis response, practice refinement, dynamic evolution, and overall adjustment in crisis learning form the basis of the pathways for policy adjustment. Finally, decision-makers aim to adapt to crisis situations by means of crisis learning while upholding stable policy beliefs amidst change.

The study also found that, in view of current social developments and realities, government departments are crucial for crisis learning and policy adjustment. At the same time, the crisis response to this outbreak demonstrates the specificity of the emergency management vocation, which requires the involvement of many disciplines (63). The response to emergencies must improve the resilience and demand of the system. To maximize the advantages of emergency response, comprehensive crisis learning requires the involvement of multiple stakeholders and extensive collaboration by the government (64).

### 4.2 Strengths and limitations

After conducting a thorough literature review, we have established a clear connection between crisis learning and policy adjustment. This paper proves this again through the combination of policy text analysis and case study analysis, which is an innovative breakthrough for previous studies. We clarify the logic and effectiveness of crisis learning in the promotion policy adjustment, and the framework we create in this study is shown to have strong practical explanatory power in empirical test. This framework will be valuable for future research. Researchers can use this framework to make an exploratory attempt to further explore how the government performs crisis learning behavior and how to better formulate policies through the results of crisis learning.

The limitation of this paper is that the framework is proposed based on a single case with the mining of government policy texts over a period of time. We have confirmed that this framework has explanatory power through many studies, but most of the crises are unique (65), and each crisis has different characteristics depending on the environment in which it occurs, the disaster-bearing capacity of the subject, and the strength of the disaster-causing factors (66). At the same time, the logic between crisis learning and policy adjustment does not always follow the framework proposed in the paper. Crisis learning by governments shows differences in the reality of complex and changing situations (67), and the process of policy adjustment will also vary according to different decision subjects and decision environments.

### 4.3 Future research and policy recommendations

Based on the results, we try to explore some of the common features and unresolved issues of crisis learning and policy adjustment in an emergency state:

First, how to quickly build an efficient learning mechanism in the emergency state? Establishing an efficient crisis learning process relatively quickly after entering a state of emergency is a crucial step toward improving the effectiveness of crisis response and the quality of crisis learning. It is worth exploring how to take the initiative to actively implement crisis learning in crisis response and better complete policy adjustment.

Second, how to make the results of crisis learning work stably in the practice of policy adjustment? The experience of crisis learning needs to be rapidly transformed into the construction of crisis management systems represented by plans, measures, and policies, and how to transform results of crisis learning from empirical lessons into policy adjustment is an area that requires further consideration.

We also try to make two policy recommendations:

First, the government should design a specific program for entering crisis learning situations. The first task is to improve an appropriate legislative condition for the for the start and operation of the crisis learning mechanism. Improving the emergency plan is an important task during the routine management (68). At the same time, it is necessary to build a team of high-level administrative personnel, strengthen the government’s sensitivity to the crisis, and improve the government’s ability to detect the crisis.

Second, government departments should pay attention to the results of learning activities. Emphasize the opinions and suggestions of experts. Attempts should be made to improve the government’s ability to deal with complex crisis situations through the application of artificial intelligence (69), big data technology (70), and the development of information platforms (71). It is recommended that the government expand the opportunities for public participation in policy formulation (72), and accelerate the establishment of an open decision-making process through institutional development.

## Data availability statement

The original contributions presented in the study are included in the article/supplementary material, further inquiries can be directed to the corresponding authors.

## Author contributions

CW: Conceptualization, Data curation, Formal analysis, Funding acquisition, Investigation, Methodology, Project administration, Resources, Supervision, Validation, Writing – original draft, Writing – review & editing. JX: Conceptualization, Data curation, Formal analysis, Investigation, Methodology, Resources, Validation, Writing – original draft, Writing – review & editing. ZX: Data curation, Investigation, Resources, Writing – review & editing.
